# CBX2 promotes cervical cancer cell proliferation and resistance to DNA-damaging treatment *via* maintaining cancer stemness

**DOI:** 10.1016/j.jbc.2025.108170

**Published:** 2025-01-08

**Authors:** Wenhan Li, Ru Shi, Yumei Gao, Xiaoman Wang, Tiantian Shen, Xiaoli Liu, Qiulei Wu, Xiaohan Xu, Zanhong Wang, Shi Du, Si Sun, Lu Yang, Jing Cai, Lin Liu

**Affiliations:** 1 Department of Obstetrics and Gynecology, Union Hospital, Tongji Medical College, Huazhong University of Science and Technology, Wuhan, China; 2 Department of Obstetrics and Gynecology, Shanxi Children’s Hospital, Shanxi Maternal and Child Health Hospital, Taiyuan, Shanxi, China; 3 Department of Gynecology, Qingdao Municipal Hospital, Qingdao, China; 4 Department of Obstetrics and Gynecology, Third Hospital of Shanxi Medical University, Shanxi Bethune Hospital, Shanxi Academy of Medical Sciences, Tongji Shanxi Hospital, Taiyuan, China

**Keywords:** PRC1, tumor proliferation, chemoresistance, radioresistance, cervical carcinoma

## Abstract

Cervical cancer is the fourth most common malignancy and the fourth leading cause of cancer-related death among women. Advanced stages and resistance to treatment in cervical cancer induce cancer-related deaths. Although epigenetics has been known to play a vital role in tumor progression and resistance, the function of epigenetic regulators in cervical cancer is an area of investigation. In this study, we focused on an epigenetic regulator, polycomb repressor complex 1 in cervical cancer. Through bioinformatics analysis and immunochemistry, we subsequently identified chromobox 2CBX2), the deregulated subunit of polycomb repressor complex 1, which is upregulated in cervical cancer and associated with poor prognosis and unfavorable clinicopathological characteristics. We provided functional evidence demonstrating that CBX2 promoted cervical cancer cell proliferation. Furthermore, CBX2 exhibited an antiapoptotic effect, which induced resistance to cisplatin and ionizing radiation in cervical cancer cells. Moreover, CBX2 was involved in maintaining cancer stemness. These findings suggest that CBX2 plays an important role in cervical cancer progression and resistance to treatment, and may serve as a potential biomarker for prognosis and resistance as well as a potential therapeutic target.

Cervical cancer is the fourth most common malignancy among women and accounted for 8.1% of cancer-related deaths among women worldwide in 2022 (1). These deaths primarily result from advanced stages of disease and are resistance to treatment. In addition to surgery for tumors confined to the uterine cervix, radiation therapy and platinum-based chemotherapy are widely used in adjuvant settings for early stage tumors, and concurrent chemoradiation (systemic administration of cisplatin plus pelvic radiotherapy) is the standard care for advanced and recurrent diseases (2). However, the response to cisplatin-based chemotherapy and radiotherapy varies among patients, and the survival rate of nonresponders has been generally inferior to that of responders (3, 4). Therefore, exploring the mechanism underlying cancer progression and resistance to treatment is essential for improving the prognosis of patients with cervical cancers.

Both cisplatin and ionizing radiation (IR) induce cell death by causing DNA damage. The fate of tumor cells exposed to DNA damage is largely determined by the subsequent DNA repair processes. Epigenetic silencing of genes mediated by histone modifications is known to favor DNA-damage repair (5, 6) and is broadly implicated in tumorigenesis (7), tumor progression (8), and the development of resistance to antitumor treatment (9, 10). Polycomb repressor complex 1 (PRC1), along with PRC2 which mediates lysine 27-methylated histone H3 (H3K27me3), contributes to the epigenetic silencing of genes by catalyzing monoubiquitination at Lys119 of histone H2A and compacting chromatin. The PRC1 complexes are composed of chromobox proteins (CBXs), polycomb group ring fingers, human polyhomeotic homologs, and E3-ligase proteins (RINGs) (11). The PRC1 complex subunits exhibit a multitude of functions, including the regulation of anoikis (12), cancer cell stemness (13, 14, 15, 16), and tumor progression (17). However, the specific roles of these subunits in the progression and treatment resistance of cervical cancer remain to be elucidated.

In this study, *via* bioinformation analysis and immunohistochemistry (IHC), we found that chromobox 2 (CBX2), rather than other PRC1 components, was significantly upregulated in cervical cancer and was associated with poor prognosis. Therefore, we focused on the expression and functions of CBX2 in cervical cancer. CBX2 has a unique AT-hook DNA binding domain compared to other CBX family members, a chromodomain (CD) with a preference for binding to lysine 27-methylated histone H3 (H3K27me3), two AT-hook-like (ATL) motifs and a polycomb repressor box (18). The CD and AT-hook modules of CBX2 are essential for its association with chromatin (18). CBX2 recognizes H3K27me3 *via* its CD and facilitates canonical PRC1 binding to chromatin, catalyzing the formation of monoubiquitinated histone H2A at lysine 119 (H2AK119ub1) which leads to the compaction of chromatin, thereby maintaining transcriptional silencing of genes. CBX2 forms condensates *via* its intrinsically disordered region (19). Moreover, the charged disordered region of CBX2 governs the phase separation of PRC1 (20). Additionally, CBX2 is involved in the formation of polycomb condensates, which can enhance epigenetic markers (21). CBX2 exists in two distinct isoforms, namely CBX2.1 and CBX2.2 which lacks polycomb repressor box domain and does not participate in PRC1 complexes (22). CBX2 has been demonstrated to play a role in the process of sexual development (23). The mutated CBX2.1 was unable to effectively regulate the downstream targets that are crucial for sexual development (24). Owing to the function of CBX2 in gene expression, CBX2 also participates in pluripotency and differentiation in stem cells (25, 26, 27).

Furthermore, CBX2 is also involved in cancer progression. Prior studies have demonstrated that CBX2 facilitates cancer cell proliferation, enhances cancer stemness, and suppresses apoptosis (12, 17, 28, 29, 30, 31). One bioinformatics analysis study revealed a correlation between CBX2 mRNA expression and the pathological stage in cervical cancer (32). Nevertheless, the specific role of CBX2 in cervical cancer remains to be elucidated owing to the scarcity of both *in vivo* and *in vitro* experimental data and the correlation between CBX2 protein levels and clinicopathological characteristics in cervical cancer patients. Here, we demonstrate the promoting effects of CBX2 on cancer cell proliferation, resistance to cisplatin and IR, and cancer stemness *in vitro* and *in vivo*, unveiling the possibility of CBX2 as a biomarker of resistance to DNA-damaging treatment and a therapeutic target in cervical cancer, which warrants further investigations.

## Results

### High CBX2 expression is associated with poor prognosis of cervical cancer patients

**Figure 1:**
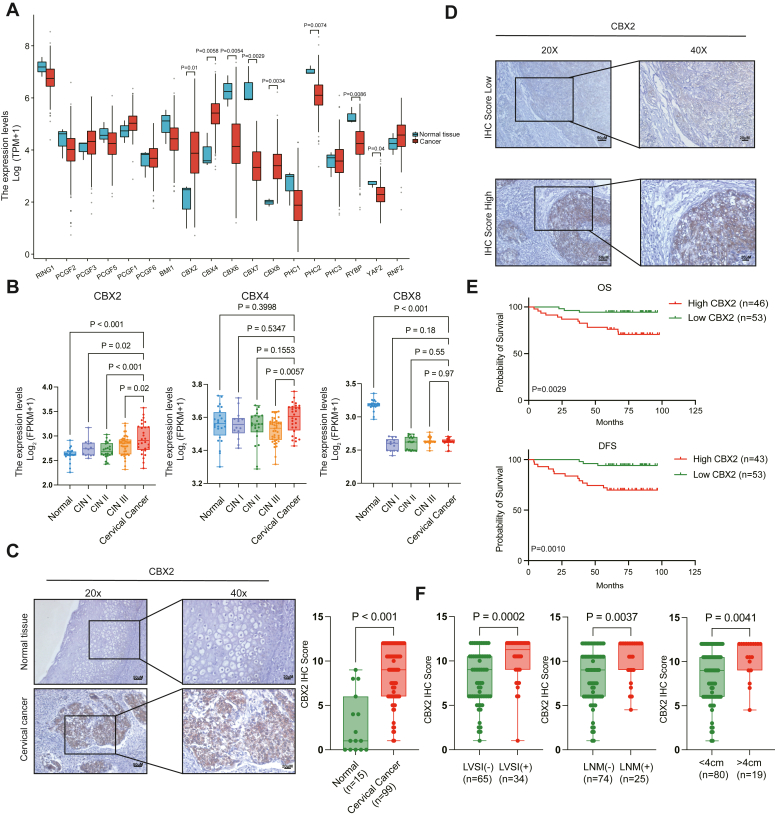
**CBX2 is upregulated in cervical cancer and associated with poor prognosis.***A*, The components of PRC1gene expression between normal tissues and cervical cancer tissues in TCGA and GTEx database. *p* values are calculated by the Mann-Whitney *U* test. *B*, The upregulated components of PRC1gene expression among normal, CIN I, CIN II, CIN III, and cervical cancer tissues in GSE63514. *p* values are calculated by one-way ANOVA. *C*, Representative images of the immunohistochemistry (IHC) of CBX2 in normal and cervical cancer tissues are on the *left*. Statistical diagrams of the IHC of CBX2 in normal and cervical cancer tissues on the right. *p* values are calculated by the Mann-Whitney *U* test. *D*, Representative images of the IHC of CBX2 in cervical cancer tissues. *E*, Kaplan-Meier curve of OS and PFS for patients in the high CBX2 expression and low CBX2 expression groups. *p* values are calculated by log-rank (Mantel-Cox) test. *F*, Statistical diagrams of the IHC score of CBX2 in LVSI (−) and LVSI (+), LNM (−) and LNM (+), tumor tissue > 4 cm and < 4 cm patients. *p* values are calculated by the Mann-Whitney *U* test. PRC1, polycomb repressor complex 1; PFS, progression-free survival; OS, overall survival; TCGA, The Cancer Genome Atlas; GTEx, Genotype-Tissue Expression; CBX, chromobox.

To identify the PRC1 components that may play a role in cervical cancer, we used the The Cancer Genome Atlas (TCGA) and Genotype-Tissue Expression (GTEx) datasets to compare the transcriptional levels of PRC1 components in cervical cancers patients with those in normal controls. We found that *CBX2*, *CBX4*, and *CBX8* presented notably increased expression, whereas *PHC2*, *CBX6*, and *CBX7* presented decreased expression in cervical cancers (Fig. 1*A*). In GSE63514, increased expression of *CBX2,* but not *CBX4* or *CBX8*, was detected in cervical cancer patients compared with normal controls, and cervical precancerous lesions were observed (Fig. 1*B*). For validation, the CBX2 protein was detected *via* IHC analysis in 99 cervical cancers and 15 normal cervix tissues. The CBX2 IHC score in cervical cancer was significantly greater than that in normal cervix, and the CBX2 signal in cervical cancer patients was predominantly located in tumor nests (Fig. 1*C*).

To assess the potential role of CBX2 in cervical cancer, we further analyzed the associations between the CBX2 levels and patient survival and clinicopathological characteristics. The 99 cervical cancers were divided into two groups (low CBX2 and high CBX2) according to their CBX2 IHC scores (Fig. 1*D*). Kaplan-Meier survival curves revealed that high CBX2 was significantly related to reduced progression-free survival (PFS) and overall survival (OS) (Fig. 1*E*). Moreover, elevated CBX2 levels were significantly associated with age ≥ 45 years, lymph vascular invasion, lymph node metastasis, and tumor size ≥ 4 cm (Fig. 1*F* and Table 1), indicating a tumor-promoting role of CBX2 in cervical cancer.

**Table 1:** The correlation between CBX2 IHC scores and clinicopathological characteristics of cervical cancer patients (*N* = 99)

Clinical pathological parameters	CBX2 IHC score		*p* value
	High	Low	
Age (years)			
<45	25 (25.3%)	16 (16.2%)	0.015
≥45	21 (21.2%)	37 (37.3%)	
Histological type			
Squamous cell carcinoma	42 (42.4%)	42 (42.4%)	0.095
Adenocarcinoma	4 (4.0%)	11 (11.1%)	
FIGO stage (2009)			
IA-IB1	27 (27.3%)	40 (40.4%)	0.075
IB2-IIA2	19 (19.2%)	13 (13.1%)	
LVSI			
Positive	23 (23.3%)	11 (11.1%)	0.002
Negative	23 (23.3%)	42 (42.4%)	
LNM			
Positive	16 (16.2%)	9 (9.1%)	0.042
Negative	30 (30.3%)	44 (44.4%)	
Tumor size (cm)			
<4	33 (33.3%)	47 (47.5%)	0.033
≥4	13 (13.1%)	6 (6.1%)	

IHC, immunohistochemistry; CBX2 IHC score high, CBX2 IHC score >9; CBX2 IHC score low, CBX2 IHC score ≤9; FIGO, International Federation of Gynecology and Obstetrics; LVSI, lymph vascular invasion; LNM, lymph node metastasis.

### CBX2 promotes cervical cancer cell proliferation

**Figure 2:**
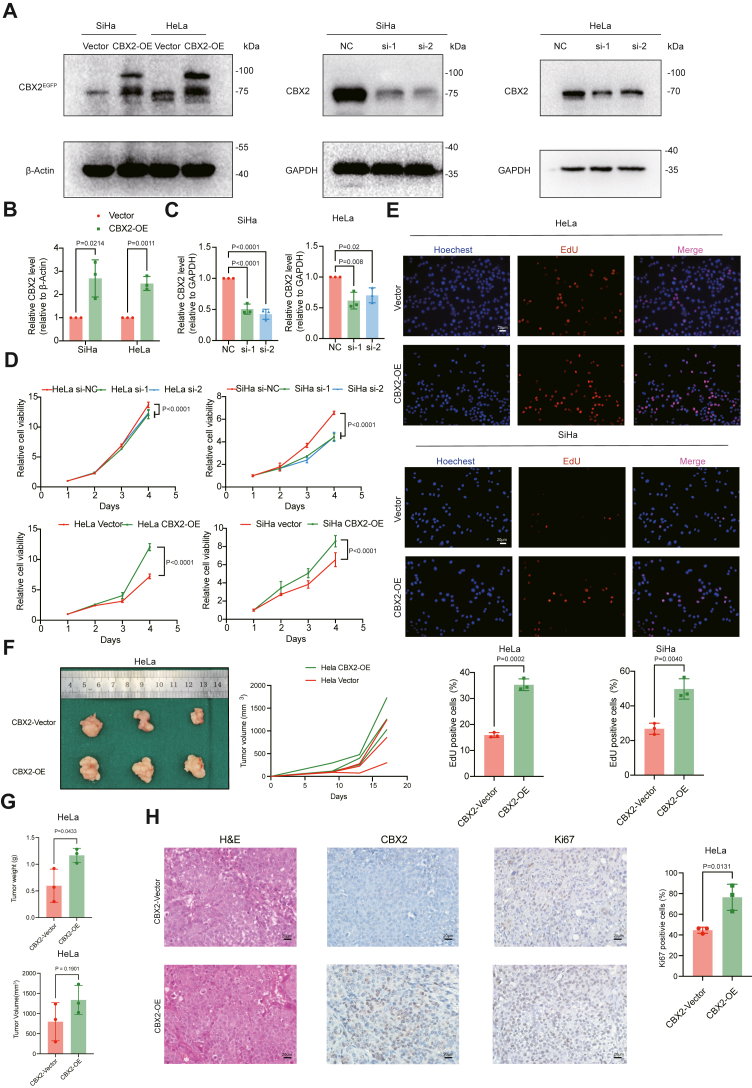
**CBX2 regulates cervical cancer cell proliferation *in vitro*.***A*, Verification of CBX2 overexpression and CBX2 knockdown in SiHa and HeLa cells. *B* and *C*, Statistical diagrams of the CBX2 overexpression and CBX2 knockdown Western blotting. Data are represented as mean ± SD. *p* values are calculated by the Student’s *t* test and one-way ANOVA. *D*, Cell proliferation curves generated from the CCK-8 assays in cervical cancer cells after CBX2 overexpression and CBX2 knockdown. Data are represented as mean ± SD. *p* values are calculated by one-way ANOVA for CCK-8 assays in CBX2 knockdown cells and the Student's *t* test for CCK-8 assays in CBX2 overexpression. *E*, Representative images of the EdU assays in CBX2 overexpression cell lines on the *top*. Statistical diagrams of the results of the EdU assays on the *bottom*. Data are represented as mean ± SD. *p* values are calculated by Student's *t* test. *F*, Images of subcutaneous xenograft tumors in nude mice in the HeLa CBX2-Vector group and CBX2-OE group on the *left*. Tumor growth curves of the HeLa CBX2-Vector group and CBX2-OE group on the *right*. *G*, Statistical diagrams of the tumor weight of the HeLa CBX2-Vector group and CBX2-OE group. Data are represented as mean ± SD. *p* values are calculated by the Student's *t* test. *H*, Representative images of the H&E of subcutaneous xenograft tumors and IHC of CBX2 and Ki67 in the HeLa CBX2-Vector group and CBX2-OE group on the *left*. Statistical diagrams of the percentage of the Ki67 positive cell of the HeLa CBX2-Vector group and the CBX2-OE group on the *right*. Data are represented as mean ± SD. *p* values are calculated by Student's *t* test. IHC, immunohistochemistry; CBX, chromobox; CCK-8, Cell Counting Kit-8; EdU, 5-ethynyl-2′-deoxyuridine.

Next, we performed functional experiments to determine the role of CBX2 in cervical cancer growth. The expression of CBX2 in the cervical cancer cell lines HeLa and SiHa cells was efficiently manipulated by transfection with lentivirus carrying CBX2-expressing vectors or small interfering RNAs (siRNAs) targeting CBX2 mRNA (Fig. 2, *A–C*). The results of the Cell Counting Kit-8 assays showed that overexpression (OE) of CBX2 significantly promoted cell proliferation and that knockdown (KD) of CBX2 inhibited cell proliferation in cervical cancer cells (Fig. 2*D*). Similarly, the results of the 5-ethynyl-2′-deoxyuridine (EdU) assays revealed notably greater percentages of proliferation cells in the cervical cancer cells overexpressing CBX2 than in the control cells (Fig. 2*E*). To further determine the role of CBX2 in tumor growth *in vivo*, HeLa CBX2-OE, and CBX2-vector cells were subcutaneously injected into BALB/C nude mice, and the tumor growth was monitored (Fig. 2*F*). The HeLa CBX2-OE tumors exhibited a higher tumor volume trend and a higher tumor weight compared to the CBX2-vector tumors (Fig. 2*G*). Moreover, the Ki67 level was considerably increased in the CBX2 OE tumors (Fig. 2*H*), indicating increased cancer cell proliferation. These results indicate that CBX2 can promote cervical cancer cell proliferation.

### CBX2 knockdown sensitizes cervical cancer cells to cisplatin

**Figure 3:**
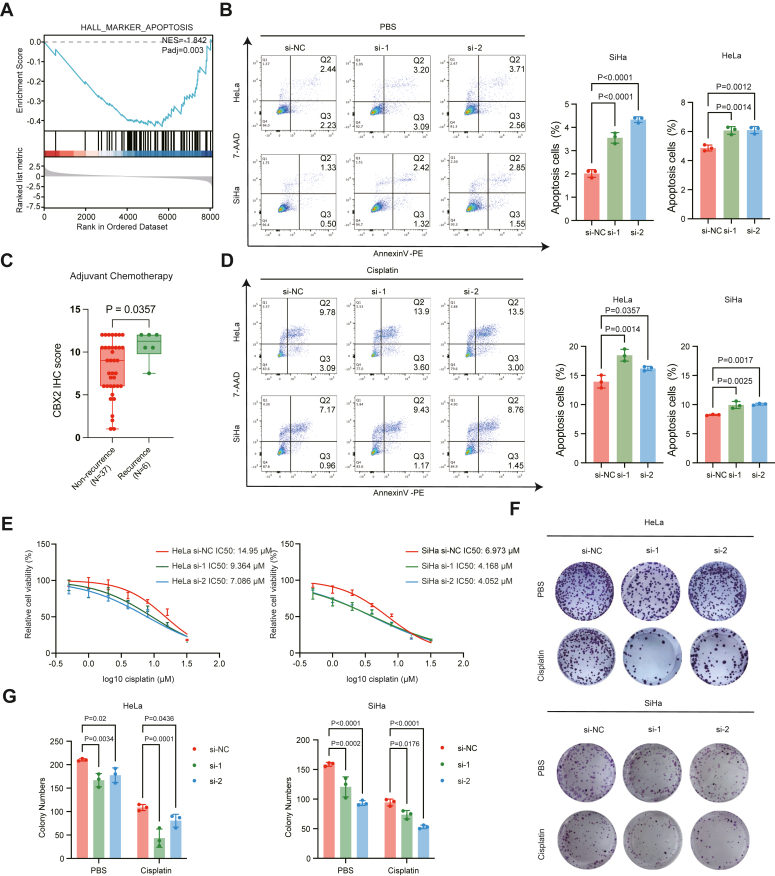
**CBX2 regulates chemoresistance in cervical cancer cells.***A*, The GSEA enrichment analysis. The Hall_marker_apoptosis is significantly enriched in the group of low *CBX2 expression* in the TCGA-CESC cohort. *B*, Representative flow cytometry results of apoptosis in SiHa cells transfected with si-NC, si-1, si-2 on the *left*. Statistical diagrams of the percentage of cell apoptosis in the SiHa and HeLa cells revealed by flow cytometry. Both early apoptotic cells and late apoptotic cells (7-AAD positive or negative and Annexin V-PE positive) are counted on the *right*. Data are represented as mean ± SD. *p* values are calculated by one-way ANOVA. *C*, Statistical diagrams of the IHC score of CBX2 in patients who received adjuvant chemotherapy. Data are represented as mean ± SD. *p* values are calculated by the Mann-Whitney U test. *D*, Representative flow cytometry results of apoptosis in HeLa and SiHa cells transfected with si-NC, si-1, and si-2 after being treated with 10 μM cisplatin for 24 h on the *left*. Statistical diagrams of the percentage of cell apoptosis in the SiHa and HeLa cells revealed by flow cytometry. The proportion of apoptotic cells (7-AAD positive or negative and Annexin V-PE positive) was compared between groups. Data are represented as mean ± SD. *p* values are calculated by one-way ANOVA. *E*, The cell viability curves show the results of MTT assays of SiHa and HeLa cells transfected with the *CBX2* siRNAs or NC siRNA treated with cisplatin for 48 h. Data are represented as mean ± SD. MTT, methyl thiazolyl tetrazolium. *F*, Representative colony formation assay images of HeLa and SiHa cells transfected with *CBX2* si-1, si-2 or si-NC 2 weeks after treatment with PBS or cisplatin (1 μM) for 48 h. *G*, statistical diagrams of (*F*). Data are represented as mean ± SD. *p* values are calculated by one-way ANOVA. IHC, immunohistochemistry; GSEA, gene set enrichment analysis; TCGA, The Cancer Genome Atlas; MTT, 3-(4,5-dimethylthiazol-2-yl)-2,5-diphenyltetrazolium bromide; CBX, chromobox; CESC, cervical squamous cell carcinoma; siRNA, small interfering RNAs.

To investigate the effect of CBX2 on the response of cervical cancer cells to cisplatin, we analyzed the degree of cell apoptosis (the major mechanism underlying the cytotoxicity of cisplatin and IR), cell viability, and colony formation ability of cervical cancer cells with CBX2 depletion (transfected with si-1 and si-2) exposed to PBS or cisplatin, compared with the negative control (transfected with si-NC). Gene set enrichment analysis revealed that the apoptosis was enriched in the cervical cancers with lower expression of *CBX2* in the TCGA database (Fig. 3*A*). In addition, CBX2 KD increased the percentage of apoptotic HeLa and SiHa cells (Fig. 3*B*). In a subgroup of patients who received adjuvant platinum-based chemotherapy (N = 43), we found that the CBX2 IHC score was significantly higher in the patients with recurrence, compared with those with nonrecurrence (Fig. 3*C*), supporting the role of CBX2 in platinum-resistance. Under exposure to 10 μM cisplatin for 24 h, the cells with CBX2 KD presented higher apoptotic percentages than did NC cells (Fig. 3*D*). Moreover, the results of the 3-(4,5-dimethylthiazol-2-yl)-2,5-diphenyltetrazolium bromide (MTT) assays revealed that CBX2 KD decreased the IC_50_ of cisplatin in both the HeLa and SiHa cell lines (Fig. 3*E*). In addition, colony formation assays revealed that CBX2 KD inhibited the clonogenic growth of cervical cancer cells treated with cisplatin (Fig. 3, *F* and *G*). These results revealed that the CBX2 KD increases the sensitivity of cervical cancer cells to cisplatin.

### CBX2 confers radioresistance in cervical cancer cells

**Figure 4:**
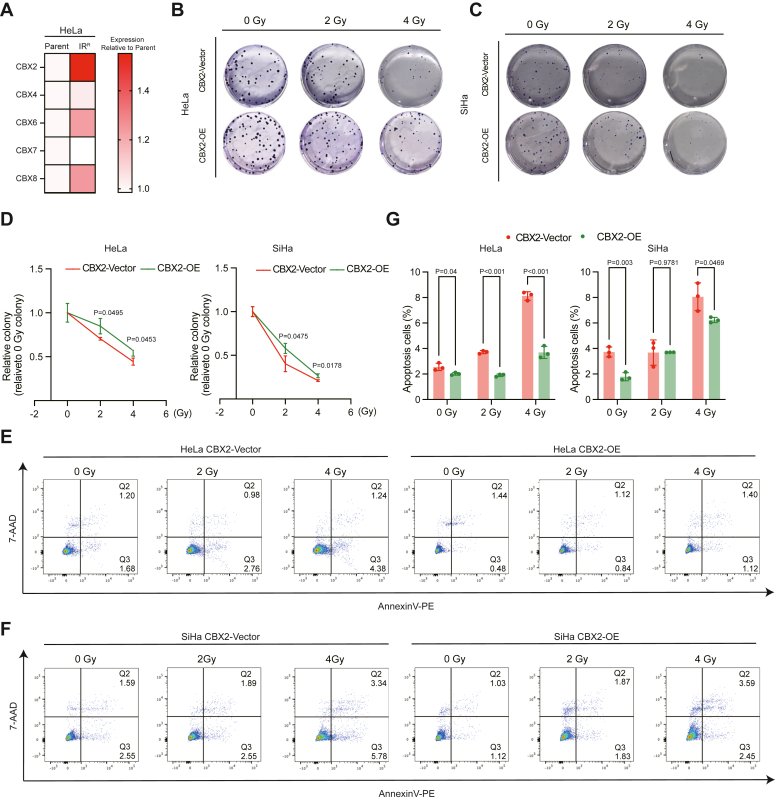
**CBX2 promotes radioresistance in cervical cancer cells**. *A*, Heatmap depicting the CBX family genes of PRC1in HeLa parent and IR resistant. Log2FC of expression compared to HeLa parent is shown. *B* and *C*, Representative colony formation assay images of HeLa CBX2-Vector and CBX2-OE, SiHa CBX2-Vector, and CBX2-OE cells 1 week after exposure under different radiation doses (0, 2, and 4Gy). *D*, Statistical diagrams of (*B*) and (*C*). Data are represented as mean ± SD. *p* values are calculated by the Student’s *t* test. *E* and *F*, Representative flow cytometry results of apoptosis in HeLa CBX2-Vector and CBX2-OE, SiHa CBX2-Vector and CBX2-OE cells 1 week after exposure under different radiation doses (0, 2, and 4Gy). The proportion of apoptotic cells (7-AAD positive or negative and Annexin V-PE positive) was compared between groups. *G*, Statistical diagrams of (*E*), (*F*). Data are represented as mean ± SD. *p* values are calculated by the Student’s *t* test. PRC1, polycomb repressor complex 1; IR, ionizing radiation; CBX, chromobox.

In parallel, we investigated whether CBX2 contributes to radioresistance in cervical cancer cells or not. We analyzed the expression of PRC1 complex subunits CBXs in IR-resistant HeLa cells and parent HeLa cells in GSE181080. Compared with parent cells, IR-resistant HeLa cells presented the highest expression of *CBX2*, followed by *CBX6* and *CBX8* (Fig. 4*A*). Furthermore, colony formation assays revealed that CBX2 OE decreased the sensitivity of HeLa and SiHa cells to 2Gy and 4Gy IR (Fig. 4, *B–D*) and resulted in a reduction in the proportion of apoptotic cells following IR exposure (Fig. 4, *E–G*). These findings demonstrate that CBX2 promotes radioresistance in cervical cancer cells.

### CBX2 maintains cervical cancer cell stemness

**Figure 5:**
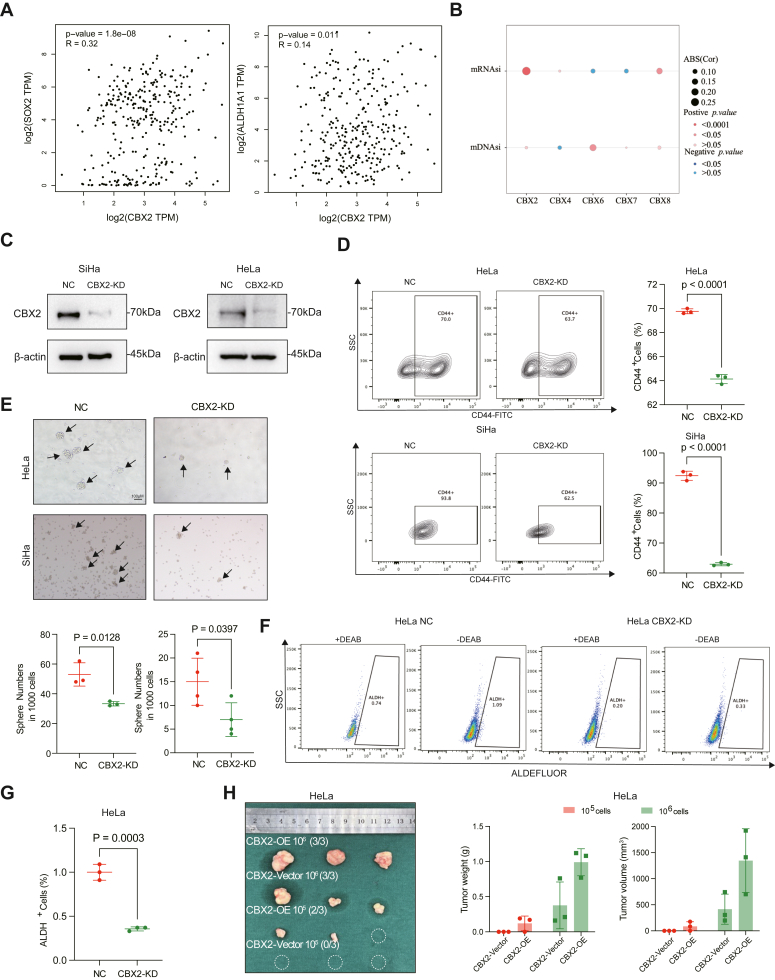
**CBX2 maintains cervical cancer stemness.***A*, Correlations between *CBX2* expression and *SOX2*, *ALDH1A1*. *B*, Correlations between CBX2, CBX4, CBX6, CBX7, CBX8, and mRNAsi. *C*, Verification of CBX2 knockdown in SiHa and HeLa cells. *D*, Representative flow cytometry results of CD44^+^ cells in HeLa NC and HeLa CBX2-KD, SiHa NC, and SiHa CBX2-KD cells on the *left*; Statistical diagrams of the results on the *right*. Data are represented as mean ± SD. *p* values are calculated by the Student’s *t* test. *E*, Representative images of sphere formation in HeLa NC and HeLa CBX2-KD, SiHa NC and SiHa CBX2-KD cells. Statistical diagrams of the results on the *bottom*. Data are represented as mean ± SD. *p* values are calculated by the Student’s *t* test. *F*, Representative flow cytometry results of ALDEFLUOR assays in HeLa NC and HeLa CBX2-KD cells. *G*, Statistical diagrams of (*F*). Data are represented as mean ± SD. *p* values are calculated by the Student’s *t* test. *H*, Images of subcutaneous xenograft tumors in nude mice in the HeLa CBX2-Vector 10^6^ cells group, CBX2-Vector 10^5^ cells group, CBX2-OE 10^5^ cells group, and CBX2-OE 10^6^ cells group. Statistical diagrams of the tumor weight and tumor volume of the HeLa CBX2-Vector 10^6^ cells group, CBX2-Vector 10^5^ cells group, CBX2-OE 10^5^ cells group, and CBX2-OE 10^6^ cells group. Data are represented as mean ± SD. CBX, chromobox; KD, knockdown; mRNAsi, a stemness index based on mRNA expression.

Tumor stem cells typically possess enhanced resistance to chemotherapy and radiotherapy. Thus, we hypothesized that CBX2 enhances the chemoresistance and radioresistance of tumor cells by maintaining stemness of tumor cells. In TCGA-cervical squamous cell carcinoma (CESC) cohort, CBX2 expression was positively correlated with the expression of the cancer stem cell markers *SOX2* and *ALDH1A1* (Fig. 5*A*). Similarly, CBX2 expression was also positively correlated with mRNAsi, a stemness index based on mRNA expression, in the TCGA-CESC cohort (Fig. 5*B*). Furthermore, we used CRISPR-Cas9 technology to knock down CBX2 in SiHa and HeLa cells (Fig. 5*C*). Flow cytometry revealed that the KD of CBX2 in cervical cancer cells resulted in decreased percentages of CD44^+^ population (Fig. 5*D*). CBX2-KD HeLa and SiHa cell formed fewer tumor spheres compared to HeLa NC and SiHa NC cells (Fig. 5*E*). Additionally, the ALDEFLUOR assay also revealed a greater percentage of ALDH^+^ cells in HeLa NC and SiHa NC cells than CBX2-KD cells. Furthermore, the tumorigenicity of HeLa OE *versus* HeLa vector cells in BALB/c nude mice was examined. The mice were subjected to subcutaneous inoculation of 10^5^ or 10^6^ cells. All the mice that received 10^6^ HeLa cells, regardless of the CBX2-OE or CBX2-vector, were able to develop xenografts. In the mice that received as few as 10^5^ tumor cells, HeLa OE cells resulted in tumor formation in two-thirds of the mice population, whereas HeLa vector cells failed to form any observable tumors (0/3, Fig. 5*H*). These results suggest that CBX2 plays a crucial role in maintaining cancer stemness.

## Discussion

The components of PRC1 are deregulated in multiple cancers, and their dysfunction is responsible for proliferation and antiapoptosis (11). Our data revealed a previously undescribed deregulated PRC1 component CBX2 in cervical cancer and showed that CBX2 plays a critical role in cervical cancer proliferation and resistance. Furthermore, these results also provide evidence that CBX2 might be a biomarker for cervical cancer prognosis and resistance.

To date, few studies have reported the deregulation of components of PRC1 and their role in cervical cancer, with the exception of BMI1. BMI1 has been reported to be upregulated in cervical cancer and promoted cancer progression and tumorigenesis (33, 34). However, *BMI1* mRNA expression levels did not differ between normal and cervical cancer tissues in TCGA and GTEx databases in our study. Post transcriptional and posttranslational modifications could account for the discrepancy of BMI1 mRNA and protein levels. Our findings indicate that the CBX family genes were more deregulated than other PRC1 components. Among these CBX family genes, *CBX7* is downregulated, and according to a previous study, CBX7 inhibits cervical cancer cell growth and induces apoptosis (35), while our data showed that the mRNA level and protein levels of CBX2 were consistently upregulated, and promoted cervical cancer cell proliferation as well as inhibited apoptosis. Although CBX proteins are thought to target PRC1 to chromatin by binding H3K27me3 (11), Glancy *et al.* reported that PRC2.1 recruited CBX2/4-cPRC1, whereas PRC2.2 recruited CBX7-cPRC1 (36). Another report indicated that competition between the PRC2.1 and 2.2 subcomplexes regulated PRC2 chromatin occupancy in human stem cells (37). When the ratio of PRC2.1 to PRC2.2 increases, PRC2 chromatin occupancy also increases. The opposing roles of the two CBX proteins may reflect the distinct roles of PRC2.1 and PRC2.2 in cervical cancer.

CBX2 is an essential regulator of cancer progression. Previous studies have shown that CBX2 promotes breast cancer cell growth (38); *CBX2* depletion inhibits the proliferation of gastric cancer (29) and colorectal cancer cells (28); and CBX2 regulates proliferation and apoptosis *via* the phosphorylation of YAP in hepatocellular carcinoma (30). These studies collectively, support our findings by demonstrating that CBX2 plays a vital role in regulating cancer cell proliferation and apoptosis in various tumors. Loss of CBX2 expression can sensitize ovarian cancer to chemotherapy (12). Consistent with these findings, our data show that it can regulate chemoresistance and radioresistance in cervical cancer. In addition, a recent bioinformatics study reported that the CBX2 mRNA level was upregulated in cervical cancer and associated with cervical cancer pathological stages (32), which is consistent with our findings. However, the CBX2 mRNA level was not significantly associated with patients' OS or PFS (32), This finding is in contrast with our results, which demonstrated an association between the CBX2 protein level and both OS and PFS. This discrepancy suggests that the protein level may be a more reliable biomarker for patient prognosis than the mRNA level alone. Additionally, the composition of our cohort (International Federation of Gynecology and Obstetrics (FIGO) stage IA-IB1, 66.7% and IB2-IIA2, 32.3%) was different from that of the TCGA cohort (International Federation of Gynecology and Obstetrics stage IA-IB1, 41.3%, IB2-IIA2, 21.7%, and III-IV, 37%). This may also account for the inconsistent results.

Platinum-based chemotherapy and radiotherapy induce cell apoptosis *via* DNA damage. Successful DNA repair is essential for cell survival, particularly in cells exposed to DNA damage. PRC1, as a vital chromatin regulator, inhibits the transcription required for DNA repair (39). However, the PRC1 subunits play distinct roles in DNA repair. Several studies have reported that BMI-1 has been shown to regulate DNA end resection and homologous recombination repair (40), whereas other studies have suggested that another PRC1 subunit, RYBP, could inhibit homologous recombination and sensitize cancer cells to poly ADP-ribose polymerase inhibitors (41, 42). Loss of CBX2 induces genome instability and deregulation of transcripts involved in DNA repair (43, 44). Our data show that CBX2 facilitates antiapoptotic effect when cancer cells are exposed to DNA damage. Nevertheless, whether its antiapoptotic effect is through regulation of DNA repair remains to be investigated.

Our results suggest that CBX2 emerged as an attractive therapeutic target in cervical cancer. However, selectively inhibiting a single CBX component is highly challenging due to the high degree of structural similarity. UNC3866 serves as a chemical probe that targets the CDs of CBX proteins for PRC1 inhibition (45). UNC3866 demonstrated specificity for CBX7 but was 12-fold less specific for other CBX proteins (including CBX2) (45). In ovarian cancer, owing to the consistent function of CBX2 and CBX7 (43), UNC3866 may be a rational choice. However, the functions of CBX2 and CBX7 are opposite in cervical cancer. These findings indicate that UNC3866 may not serve as an ideal CBX2 inhibitor for cervical cancer therapy. A recent study reported that one CBX2 inhibitory peptide can inhibit ovarian cancer cell proliferation and tumor progression *in vivo* (46). This inhibitory peptide may be an option for targeting CBX2 therapy in cervical cancer.

However, this study also has several limitations. We mainly focused on the function of CBX2, but we did not validate whether this function occurred through PRC1 or not. Additionally, our results suggested that CBX2 could promote cancer cell proliferation both *in vivo* and *in vitro*. Nevertheless, although CBX2 was verified *via* different assays to promote resistance to DNA damage therapy *in vitro*, our study lacked data on animal experiments to assess the resistance *in vivo.*

In conclusion, our results identified a deregulated PRC1 component, CBX2 and established an association between CBX2, tumor progression, and resistance to DNA damage treatment, providing some evidence for future targeted therapy against CBX2 in cervical cancer.

## Experimental procedures

### Public data analysis

RNA-seq data of cervical cancer and normal cervix were downloaded from TCGA database and the GTEx database and GSE63514 was downloaded from the Gene Expression Omnibus database to identify the canonical PRC1 components expression between cervical cancer and normal cervix. In addition, GSE181080 was downloaded from Gene Expression Omnibus database (https://www.ncbi.nlm.nih.gov/geo/) to identify the CBX family genes (*CBX2*, *CBX4*, *CBX6*, *CBX7*, and *CBX8*) expression between IR resistant HeLa cells and parent HeLa cells. Gene set enrichment analysis was performed by clusterProfiler (v3.14.1, https://github.com/YuLab-SMU/clusterProfiler). The correlation between CBX2 and the stemness markers SOX2 and ALDH1A1 was analyzed by GEPIA2 (http://gepia2.cancer-pku.cn/). The mRNAsi, a stemness index based on mRNA expression, in TCGA-CESC was obtained at https://bioinformaticsfmrp.github.io/PanCanStem_Web/.

### Patients and specimens

This study complied with the Declaration of Helsinki and was approved by the Ethics Committee of Tongji Medical College, Huazhong University of Science and Technology (IORG No: IORG0003571). A total of 99 cervical cancer samples and 15 normal cervix samples from our paraffin block archives were used for CBX2 IHC staining. All the specimens were taken from patients who received surgery between January 1, 2013 and January 1, 2017 at Wuhan Union Hospital. The normal tissues were obtained from patients undergoing hysterectomy because of benign disease of the uterus, and the cancer tissues were obtained from patients with pathologically diagnosed primary cervical cancer. Patients who had other malignant diseases, conditions associated with abnormal immunity or hematopoietic function, or prior antitumor treatments were excluded. Clinicopathologic features of patients were collected from medical records, and the patients with a follow-up period of more than 6 months were included in the survival analysis.

### Immunohistochemistry

Immunohistochemistry assays were performed to detect CBX2 protein levels in human cervical tissues and xenografts, and Ki67 levels in xenografts. Briefly, paraffin slides were dehydrated through a graded alcohol series, followed by antigen retrieval in 0.01 M citrate buffer (pH = 6.0) for 20 min at 95 °C. Then, the slides were incubated with 3% H_2_O_2_ for 20 min to inactivate endogenous peroxidase and blocked with 5% goat serum for 20 min at 37 °C. Next, the slides were incubated with a primary antibody overnight at 4 °C. Horseradish peroxidase-conjugated secondary antibody was incubated for 1 h at room temperature. Furthermore, 3,3′-diaminobenzidine detection, hematoxylin, dehydration in graded ethanol, and mounting were applied. The CBX2 IHC results were scored according to the area of staining (0 = none, 1 = less than 25%, 2 = between 25% and 50%, 3 = between 50% and 75%, and 4 = 75%–100% of stained cells) multiplied by the intensity of staining (0 = none, 1 = weak, 2 = moderate, and 3 = strong). Two experienced pathologists evaluated scores in a blinded fashion. The median staining score of nine was used as the cutoff to define high and low CBX2 protein levels. Thus, patients with different levels of expression were divided into low- and high-staining groups. Antibodies used in IHC are listed in Table S1. Ki67 levels were calculated by the following formula: Ki67-positive cells = Ki67-positive cell count/(hematoxylin-positive cell count) × 100%.

### Cell culture and transfection

HeLa (cervical adenocarcinoma, CADC) and SiHa (cervical squamous cell carcinoma, CSCC) cell lines were obtained from the China Center for Type Culture Collection (Wuhan University). The cell lines used in the experiments were authenticated *via* short tandem repeat profiling at Shanghai Biowing Applied Biotechnology Co., LTD. The cells were cultured in RPMI-1640 supplemented with 10% fetal bovine serum (GIBCO), 100 units/ml penicillin, and 100 μg/ml streptomycin at 37 °C with 5% CO_2_. *CBX2* siRNAs were purchased from TsingKe Biological Technology Company: NC siRNA (si-NC, sense strand, UUCUCCGAACGUGUCACGU; antisense strand, ACGUGACACGUUCGGAGAA), CBX2 siRNA-1 (si-1, sense strand, CCAUCGUGCACUACAUGAATT; antisense strand, UUCAUGUAGUGCACGAUGGTT), CBX2 siRNA-2 (si-2, sense strand, GGCUGGUCCUCCAAACAUATT; antisense strand, UAUGUUUGGAGGACCAGCCTT). Transfection was performed using Lipofectamine 3000 (Invitrogen) according to the manufacturer's protocol. The *CBX2*-overexpressing HeLa and SiHa cells (HeLa CBX2-OE and SiHa CBX2-OE) were constructed using the lentivirus expressing *CBX2*-enhanced green fluorescent protein) fusion cDNA (GeneChem), and an EGFP-lentiviral vector was used as a NC (HeLa CBX2-Vector and SiHa CBX2-Vector). The CBX2 KD HeLa and SiHa cells (HeLa CBX2-KD and SiHa CBX2-KD) were constructed using CRISPR-Cas9 system (Genechem). The CBX2-targeting small-guide RNA sequence was 5′-CACCGGAGCTTGGAGCGCCGGCTGC-3′, and the NC small-guide RNA sequence was 5′-TTCTCCGAACGTGTCACGT-3′ (HeLa NC and SiHa NC).

### Western blot analysis

Total cellular proteins were extracted with NP40 (Beyotime Biotechnology) lysis buffer containing proteinase inhibitor cocktail (APExBIO), centrifuged at 12,500*g* for 15 min, and quantified *via* a BCA assay kit (Beyotime Biotechnology). Samples were separated by 10% SDS-PAGE and transferred onto polyvinylidene difluoride membranes. The membranes were then blocked with 5% nonfat milk in Tris–buffered saline with Tween 20 for 2 h at room temperature and incubated with primary antibodies at 4 °C overnight. The total protein extraction and Western blotting procedures were performed as previously described. Antibodies used in Western blotting are listed in Table S1.

### Cell proliferation assays

Cervical cancer cells were seeded into 96-well plates (1×10^3^ cells/well) and Cell Counting Kit-8 at a final concentration of 10%; HY-K0301, (MedChemExpress) solution was added at day 1, day 2, day 3, and day 4. After being further incubated for 4 h at 37 °C, absorbance at 450 nm was measured with a microplate reader (MD, i3x), and cellular viability fractions were calculated by normalizing the absorbance of day 2, day 3, and day 4 to that of day 1. An EdU cell proliferation assay was performed to estimate the proliferation rate of cervical cancer cells according to the instructions of BeyoClick EdU kit (C0078S). The percentage of EdU-positive cells is calculated by the following formula: the percentage of EdU-positive cells = EdU-positive cell count/(4′,6-diamidino-2-phenylindole-positive cell count) × 100%.

### Animal experiments

The animal experiment was supervised and approved by the Institutional Animal Care and Use Committee of Tongji Medical College, Huazhong University of Science and Technology. For tumor growth experiments, 4-week-old female BALB/c-nu mice were randomly divided into two groups (3 mice in each group) and received subcutaneous injections of 2 × 10^6^ HeLa CBX2-Vector or CBX2-OE cells. The tumor size was first measured on day 9 and monitored every 4 days. For tumorigenicity experiments, 4-week-old female BALB/c-nu mice were randomly divided into four groups (3 mice in each group). The HeLa CBX2-Vector and CBX2-OE cells were diluted to a density of 10ˆ5 or 10ˆ6 cells per injection. The cells were implanted subcutaneously into the right side of the interscapular region of mice. The animals were sacrificed by cervical dislocation when the largest subcutaneous tumor volume reached 2000 mm^3^. The volume of the tumor was calculated with the following formula: length × width × width/2 (mm^3^).

### MTT assay

Cervical cancer cells were seeded onto 96-well plates at a density of 5000 cells per well. The cells were then exposed to various concentrations of cisplatin for 48 h to evaluate the cytotoxicity of cisplatin to cervical cancer cells. MTT, at a final concentration of 10% (Servicebio) was then added to each well and incubated further for 4 h at 37 °C, before dimethyl sulfoxide (Biosharp) was added to dissolve the MTT formazan product. Absorbance at 570 nm was measured with a microplate reader (MD, i3x), and cellular survival fractions were calculated by normalizing the absorbance of cisplatin-treated wells to that of untreated controls. The dose-response curves were plotted by using GraphPad Prism (version 9.0.0, GraphPad Software, USA, https://www.graphpad-prism.cn). Each experiment was performed with at least three biological replicates.

### Colony formation assay

Cervical cancer cells were seeded onto 6-well plates at 1000 cells per well and incubated with cisplatin (1 μM) for 48 h. After further incubation with a medium free of drugs for 2 weeks, the colonies were fixed with 4% paraformaldehyde for 30 min, stained with 0.5% crystal violet for 30 min, and photographed. Cervical cancer cells were seeded onto 6-well plates at 1000 cells per well and exposed under different radiation doses (0, 2, 4, and 8Gy). After further incubation with a new medium for 1 week, the colonies were fixed with 4% paraformaldehyde for 30 min, stained with 0.5% crystal violet for 30 min, and photographed. The number of colonies (>50 cells) was counted using ImageJ 1.80 software (https://imagej.net/ij/). Each experiment was performed in at least three biological replicates.

### Apoptosis analysis

The cancer cells were treated with cisplatin (20 μM) for 24 h, and PBS was used as solvent control for cisplatin; the cancer cells were exposed to different radiation doses (0, 2, 4, and 8Gy). Then, the cell apoptotic rate was measured by using an Apoptosis Detection Kit (BD, 559763) on a flow cytometer (BD, LSRForstessa X-20 Special Order Product). The percentage of apoptotic cells was determined by the percentage of Annexin V positive cells. Each experiment was performed in at least three biological replicates. FlowJo software (10.4.1, https://www.flowjo.com) was used for all data analyses.

### Sphere formation assay

Cells were resuspended and placed into the 6-well ultralow plate in serum-free DMEM/F12 medium (GIBCO) supplemented with 20 ng/ml hEGF (R&D), 20 ng/ml hFGF (R&D), and 2% B27 supplement (BasoMedia, S440J7) for 5 to 7 days at 37 °C with 5% CO2. The stem cell medium was supplemented every 3 days, and the spheres were counted on the last day.

### Flow cytometry

Flow cytometry was used to detect stem cell markers CD44 (FITC Anti-Human/Mouse CD44 Antibody [IM7], Elabscience, E-AB-F1100C) in cervical cancer cells. The ALDEFLUOR Kit (STEMCELL Technologies, catalog no. 01700) was used to determine acetaldehyde dehydrogenase (ALDH) activity in cervical cancer cells following the manufacturer's instructions. Then, the CD44^+^ cell rate and ALDH^+^ cell rate were measured on a flow cytometer (BD, LSRForstessa X-20 Special Order Product). Each experiment was performed in at least three biological replicates. FlowJo software (10.4.1) was used for all data analyses.

### Quantification and statistical analysis

GraphPad Prism 9 software was used for statistical analysis, IC_50_ calculations, and survival analysis. Statistical significance was determined by Student’s *t* test, log rank (Mantel-Cox) test, Mann-Whitney test, and one-way ANOVA, as appropriate. Significance was defined as *p* < 0.05. All data were taken from at least three independent experiments.

## Data availability

This paper analyzes existing, publicly available data. The accession numbers for the datasets are listed. Any additional information required to reanalyze the data reported in this paper is available from corresponding author upon request.

## Supporting information

This article contains supporting information

## Ethics approval and consent to participate

This study complied with the Declaration of Helsinki and was approved by the Ethics Committee of Tongji Medical College, Huazhong University of Science and Technology (IORG No: IORG0003571). All study participants provided written informed consent to participate.

## Patient consent for publication

Written informed consent for publication of clinical details was obtained from all patients.

## Conflict of interest

The authors declare that they have no conflicts of interest with the contents of this article.
