# Supplementary material for: CBX2 promotes cervical cancer cell proliferation and resistance to DNA-damaging treatment via maintaining cancer stemness
Source: J Biol Chem. 2025 Jan 8;301(2):108170. doi: 10.1016/j.jbc.2025.108170 (PMC11835617; doi:10.1016/j.jbc.2025.108170)
Supplement: Table S1 [file mmc1.docx]

**Supplementary Table**

**Antibody used in this research**

| Antibody |  | Source |  | Identifier |  | Application |
| --- | --- | --- | --- | --- | --- | --- |
| CBX2 Polyclonal antibody |  | Proteintech |  | 15579-1-AP |  | WB, IHC |
| CBX2 Monoclonal antibody |  | ThermoFisher |  | **MA5-38465** |  | IHC |
| Ki67 Polyclonal antibody |  | Proteintech |  | 27309-1-AP |  | IHC |
| GAPDH Monoclonal antibody |  | Abclonal |  | A19056 |  | WB |
| β-actin Monoclonal antibody |  | Abclonal |  | AC038 |  | WB |

WB: western blotting; IHC, immunohistochemistry.
